# First surgical experience with a COVID-19 positive patient in Costa Rica: case report, staff safety protocol and brief review of literature

**DOI:** 10.1186/s40792-020-01054-x

**Published:** 2020-11-06

**Authors:** Alfredo Sanchez-Betancourt, Pablo Sibaja-Alvarez, Milagros Gonzalez-Cole, Ivannia Mendez-Barboza, Crishtna Ledezma-Cruz, Priscilla Vargas-Paez

**Affiliations:** 1 Centro de Investigacion Clinica de Oriente, Escazu, San Jose Costa Rica; 2 Hospital Mexico, Caja Costarricense del Seguro Social, La Uruca, San Jose Costa Rica

**Keywords:** Case report, COVID-19, Global health

## Abstract

**Background:**

The safe management of patients with COVID-19 has been a challenge during the current pandemic, leading to healthcare workers being disproportionately affected by the virus. In Costa Rica, 20% of all infections and 27% of all ICU cases during the initial weeks of the outbreak were healthcare workers. The existing recommendations and protocols on how to care for an infected patient that requires acute surgical management have been applied successfully in various scenarios. We look to describe the first case of a COVID-19 patient that required surgical management in Costa Rica and present a summary of the protection measures utilized in a tertiary care hospital.

**Materials and methods:**

A review of literature utilizing Embase, Medline Complete and Google Scholar was performed. A surgical case report using the SCARE statement guidelines was drafted and a brief summary of the 54 items contained in the hospital’s COVID-19 surgical protocol is described.

**Case presentation:**

We present the case of a 29 year old obese male who had acute appendicitis with perforation that contracted SARS-CoV-2 and became symptomatic at home on pod#3, who later required multiple surgeries to address an infected abdominal hematoma both while having an active COVID-19 infection and afterwards.

**Conclusions:**

Safety measures for both staff and patients are of the utmost importance during the current coronavirus pandemic. Limitations in the availability of personal protection equipment as well a lack of knowledge and experience with handling surgical patients with this condition have led to various safety and attention protocols being drafted. The successful management of this patient is the first experience in Costa Rica on how to properly address staff safety during a surgical procedure. None of the workers involved in care of this patient were diagnosed with SARS-CoV-2.

## Background

In the final months of 2019, 41 patients in the city of Wuhan, in central China were diagnosed with atypical pneumonia caused by a new strain of coronavirus[1, 2], which has a 93.3% nucleotide identity similarity to the RmYN02 strain of a bat coronavirus [3]. The first cases were associated with the wet market in the center of the capital of Hubei province[4]. The disease quickly spread beyond the locality in which it was first detected, and by January 30th, the infection by the severe acute adult respiratory syndrome coronavirus 2 (SARS-CoV-2), had been declared a global health emergency by the World Health Organization, which on March 11th upgraded the state to a global pandemic [5]. In Costa Rica (CR), the first cases were detected on March 6th[6]. Both cases were from a couple from New York city, who were visiting the country. The spread of the disease was initially slow, reaching the 1000th confirmed case by May 28th, with only 10 fatalities in that same time period.

Healthcare workers have been designated as a high-risk group for contracting and spreading the SARS-CoV-2 leading some to suggest that it should be cataloged as a work-related disease in this population [7]. In Italy, this group has made up 10% of the detected cases of Coronavirus Disease-19 (COVID-19) [8], and in some areas, around 20% of the healthcare workforce was infected [9]. This trend is in line with the infected population in CR, in which 20% of all infections and 27% of cases managed in the Intensive Care Unit (ICU) are healthcare workers. The need for proper measures to prevent the spread of the disease among medical personnel is critical. There have been multiple documented shortages of personal protection equipment (PPE) [10], this, in addition to prolonged exposure to the infectious agent and limitations in social distancing create potentially unsafe workplaces for those caring for COVID-19 patients [11]. In regards to surgeons and operating room personnel, guidelines have been set forth that look to mitigate the risk of infection when providing care to a COVID-19 positive patient that has underlying surgical pathology[12].

In this article we report the case of a 29-year-old male who had abdominal sepsis postoperatively as the result of a prior surgery due to acute appendicitis with perforation, who had been diagnosed with COVID-19 and was being cared for in the Coronavirus Center. The patient required two emergency surgeries to successfully address his intraabdominal condition. This paper also looks to summarize the local prevention methods utilized to safely manage an active COVID-19 patient that requires surgical management in the operating room (OR).

## Materials and methods

A review of literature utilizing Embase, Medline Complete and Google Scholar was performed. A surgical case report using the SCARE statement guidelines was drafted. An IRB authorization was not requested as no identifying patient information is disclosed in the article. A brief summary of the 54 items contained in the hospital’s COVID-19 surgical protocol is described.

## Case presentation

A 29-year-old obese male, who lives in the coastal town of Limon, who originally consulted on the 2nd of May for right lower quadrant pain, who was documented to have signs of peritoneal irritation, a positive McBurney and Rovsing sign, and who intraoperatively was diagnosed with an acute appendicitis with perforation and released from the local hospital after an open appendectomy and abdominal cavity washout the day after in stable condition. On May 5th, the patient consults due to cough, mild dyspnea and fever. He was tested for SARS-COV-2 and told to self-isolate in his home until the results from the nasopharyngeal swab were obtained. On May 9th, the results come back positive. Two days later, due to continuing fevers and general malaise, the patient is started on hydroxychloroquine at a dose of 400 mg q/12 h for 5 days.

**Fig. 1:**
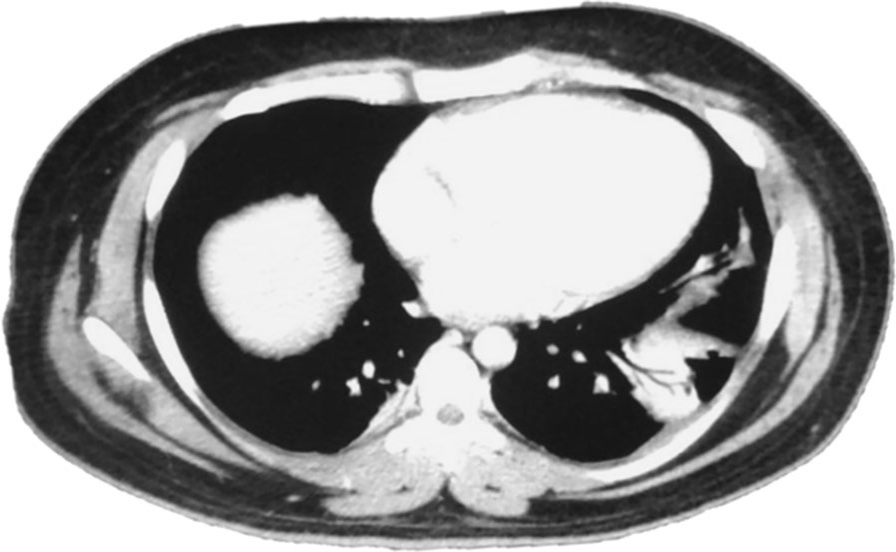
Chest CT showing atelectasis and a left sided pleural effusion

**Fig. 2:**
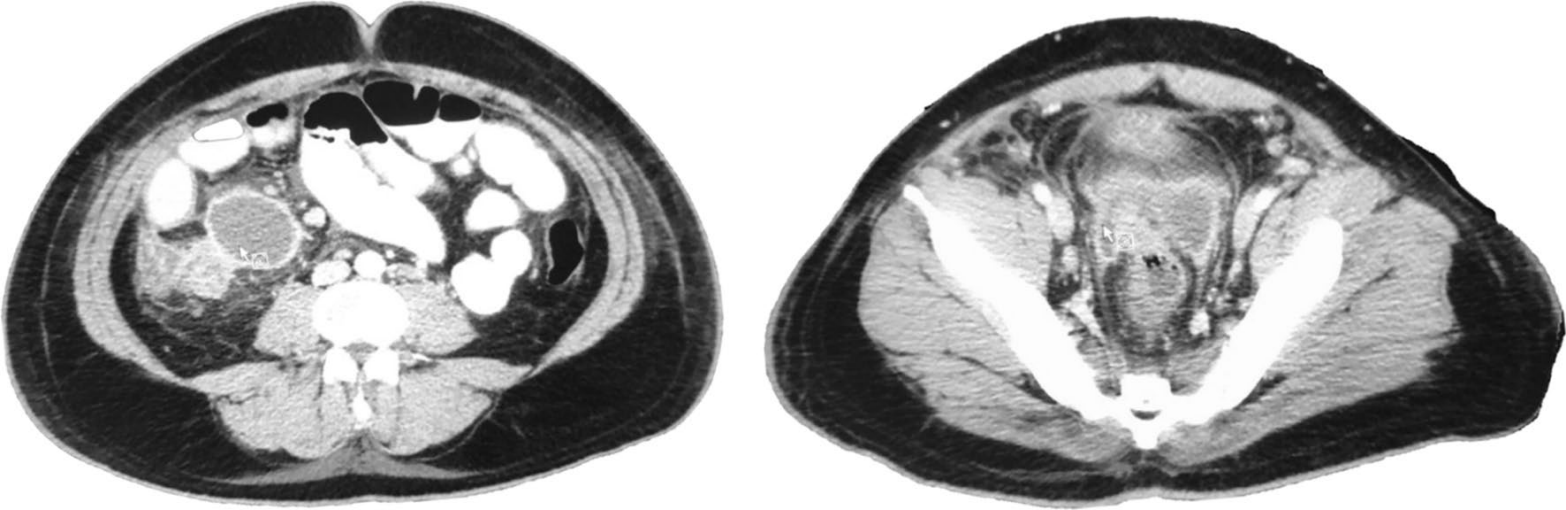
Abdomino-pelvic CT. Interloop edema, perivesical fluid infiltration and scant free fluid in the abdomino-pelvic cavity

On May 21st, the patient is seen in the ER of Limon hospital where he is evaluated for fever, moderate dyspnea, tachypnea, right lower quadrant pain and general malaise. An abdominal, pelvic and chest computerized tomography (CT) are performed. Imaging shows low grade bilateral atelectasis, unilateral pleural effusion and an abdominal fluid collection of approximately 570 cc (Figs. 1, 2). Laboratory test show a leukocytosis of 18.070 white blood cells per field with increased ferritin, D dimer and reactive thrombocytosis. A nasopharyngeal swab was collected and was positive. The patient is started on Cefotaxime and enoxaparin and transferred to the National COVID Center in capital city of San Jose.

On May 22nd, the general surgery service in the adjacent Hospital Mexico is consulted due to the abdominal collection and it is determined that surgical drainage is required. The patient had an exploratory laparotomy which retrieved approximately 500 ccs of haemopurulent fluid in the interloop space of the caecum and ascending colon which culture a multisensitive *E. coli and E. Faecalis*. The procedure had an operating time of 34 min. The patient was returned postoperatively to the National COVID Center where his antibiotics were modified to include metronidazole and vancomycin. The transfer time from the COVID center to the OR was not measured.

On May 23 and 26th the patient had two negative nasopharyngeal swabs and was declared cured of COVID-19. On the 27th, he once again presented with fevers, abdominal pain and vomiting, he was therefore discharged for the COVID center and transferred to the surgical service of Hospital Mexico where on abdominal CT another fluid collection of approximately 500 cc was observer. On May 29th (pod#27 appendectomy, pod#7 infected hematoma drainage) a diagnostic laparoscopy that was converted into an open laparotomy yielded an interloop adhesion as well as a ceacal plastron with an additional turbulent hematic fluid collection in the paracolic space. The patient was managed with an open abdomen and negative pressure wound therapy. His antibiotic coverage was updated to ertapenem and ampicillin. On June 3rd the patient is taken once again to the OR for abdominal washout and fascia closure. The patient finished his IV antibiotics on the 7th of June and was discharged in good condition the following day.

## Discussion

### Situation in Costa Rica

The SARS-COV-2 was first detected in Costa Rica more than 90 days after it was first identified in China. Initial structured and coordinated efforts to mitigate the spread of the disease appear to be successful, with lower death and transmission rates than countries with similar population sizes, gross domestic product and healthcare coverage[13]. Initial measures included suspending all mass gatherings, closing schools, places of worship, public buildings, hotels, bars, casinos and instituting a sanitary restriction on private transportation while encouraging the population to shelter in place. Easter festivities were also banned, as well as access to the beaches and national parks. There was also a closure of the country’s borders, with special emphasis placed on the northern border with Nicaragua, where no containment measures were taken by that country’s government[14], and reliable data has not been available as to the scale of the outbreak. Nicaragua is the only country in Central America not to declare a state of emergency in relation to the COVID-19 outbreak. All international airports in CR have been closed with only cargo and repatriation flights taking place since mid-March with a tentative reopening set for August 1st.

As of June 23rd, there were 2368 confirmed cases, consisting of 1049 females (44.3%) and 1319 (55.7) males. 652 positives cases were foreigners (24.7%) and 1716 (72.3%) were locals. A total of 12 deaths are attributed to COVID-19 in Costa Rica, with a current mortality rate of 0.506%. Most cases are distributed in the capital city and in the largest settlement in the north of the country, San Carlos. In the last 4 weeks there has been a doubling of cases, with a large increase in the northern part of the country. As a measure to increase hospital beds available to treat patients with COVID-19 the National Rehabilitation Hospital was transformed into the countries only COVID center.

All elective surgeries, barring oncology, have been postponed, creating an additional backlog of 22,647 surgical procedures[15]. Three major hospitals closed their outpatient consults due to the outbreak, transferring some of their medical services to teleconferencing or telephone consultations. Due to the shelter in place measures, some communicable disease’s incidence dropped during the initial phase of the COVID-19 pandemic, such as infectious diarrhea, that had a 28% year to year decrease[16].

### Local COVID-19 surgical protocol

The burden on healthcare workers during this pandemic has been high[11]. As part of the in-hospital staff, surgeons and surgical personnel have to follow previously defined regulations with regards to limiting and controlling the exposure to patients who are suspected of having COVID-19 or have been confirmed as infected, as to diminish the likelihood of infection to staff and patients. Deferrals and postponements for many types of surgeries have been a widely applied method to limit the exposure to COVID-19 not only to patients, but to healthcare workers as well[17]. Other more traditional methods of reducing exposure have been applied, with the structures use of surgical suite disinfections and strict use of PPE. A locally created protocol utilizing the recommendations emanating from the experiences and guidelines from other parts of the world[18–22] was used in the successful and safe intervention of the first COVID-19 positive patient to require surgery in Costa Rica.

The guideline divides the surgical process into three phases: preoperatory (logistics and planning) surgical and postoperative.

In the first phase, as recommended by Wong et al.(18), prepackaged surgical supply kits are created and stored inside a specially designated OR, in which all patients diagnosed or suspected of having COVID-19 will be operated on. There are signs posted on the door indicating that this is a COVID-19 surgical room. All nonessential items for the surgery should be removed from the OR prior to the initiation of the COVID-19 surgical protocol. Two surgical teams are designated, A notification is sent out to the head of each surgical team prior to the surgery being scheduled. The teams consist of one nurse, one nursing assistant, a surgical technologist, an anesthesiologist and two surgeons. No additional personnel can enter. One of the teams enter the COVID-19 OR prior to the surgery while the other team is on stand-by, with support staff of said team being tasked with logistical duties in case the need arises for additional equipment or medications during the procedure. The anesthesiologist inside the OR is responsible for all the supplies and equipment inside the OR, with special care being placed on the videolaryngoscope, which must be checked and made operational prior to the patient being admitted into the OR. The OR’s ventilation system is placed on negative pressure.

**Fig. 3:**
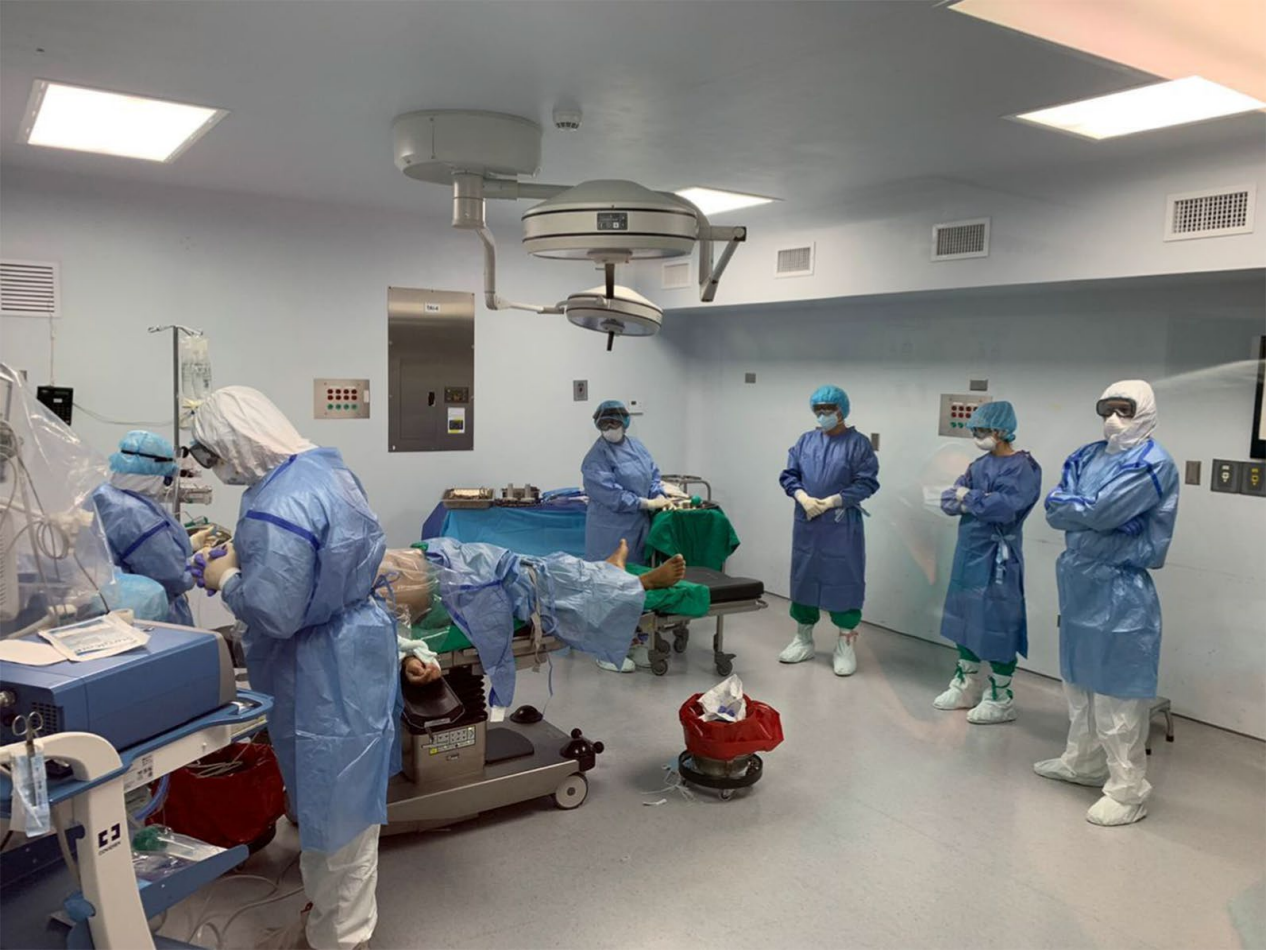
COVID-19 surgical team wearing the recommended PPE during the initial part of the operation

All surgical personnel must wear the appropriate PPE (Fig. 3), which consists of a N95 mask, safety goggles with or without a face shield, a biohazard suit, disposable waterproof boot covers and two pair of sterile globes (nitrile and latex).

Prior to the patient being transported to the OR, an N95 mask is placed on the patient and only removed prior to intubation and is placed on the patient immediately after the surgical procedure. During endotracheal intubation, a clear plastic drape is placed over the top third of the patient, as to provide an additional barrier. Standard patient monitoring is done during the procedure.

After the surgery is performed, the patient is not transported to the regular recovery room, but is instead placed in a special biohazard room, where the second team is charged with continuing care. The first team, initiates decontamination measures once the patient leaves the OR. The part of decontamination involves taking the anesthesia machine canister and immersing it in an enzymatic cleaning solution for 20 min and disposing of all contaminated foam and gauze in the biohazard labelled trash bags that are then triple-sealed. The surgical instruments are placed in a double bin with enzymatic cleaner that is placed by the door of the OR, to be later taken to be sterilized. The technical staff then proceeds to clean the OR for 25 min using chlorhexidine soap and water first, and 0.1% chlorine solution last.

The medical and technical staff then begin removal of the PPE in pairs using a 21-step process, this is done to guarantee that the process is applied under direct supervision. Once all the PPE has been successfully removed, all staff is instructed to shower.

## Conclusions

The SARS-COV-2 pandemic has had far-reaching consequences. The economic, political, cultural and healthcare consequences have been significant. Healthcare workers are especially at risk of contracting the disease and have been drastically affected by the outbreak. The lack of preparation of foresight of all healthcare system around the world have caused severe limitations in the availability of resources to manage this pandemic. The scarcity of PPE as well as the inexperience in dealing with this kind of disease have led to many frontline workers being infected all around the world.

It is of the utmost importance that surgical staff be successful in implementing safety protocols in these perilous times. This allows for adequate surgical attention for the patients and ensures continuous care of surgical pathology. Success is based on three elements: a clear safety protocol based on the best available evidence adapted to the scenarios in which it is to be employed. A top-down command structure to ensure proper deployment and supervision of safety protocols and an experienced surgical team that is adequately trained and can limit the psychological effects of working in a stressful and dangerous environment. The integration of these three characteristics into a surgical team will allow for uninterrupted care for surgical patients while limiting staff depletion, infection and burnout.

The effects on surgical practices by the current pandemic have been widespread. A large amount of procedures were cancelled due to the outbreak, leaving healthcare services with much larger waiting lists and having the need to prioritize care in order to reduce the biological risk to both patients and staff. The necessity of treating patients with acute surgical pathology that might be or are infected with SARS-COV-2 have led to various protocols and recommendations being issued. In the hospital where this patient was managed, a local guide was created utilizing these documents. The patient was successfully operated on with none of the staff that took part in the procedure having been infected because of providing care to an infected individual.

This first experience in surgically intervening a COVID-19 patient has yielded a valuable local experience, serving as an example for further procedures that may allow staff to safely take part in the care of patients with this condition.

## Data Availability

No additional data exists.

## Abbreviations

COVID-19: Coronavirus Disease 19
CR: Costa Rica
CT: Computerized tomography
ICU: Intensive Care Unit
OR: Operating room
PPE: Personal protection equipment
POD: Postoperative day
SARS-CoV-2: Severe acute adult respiratory syndrome coronavirus 2
